# Levels and determinants of complementary feeding based on meal frequency among children of 6 to 23 months in Bangladesh

**DOI:** 10.1186/s12889-016-3607-7

**Published:** 2016-09-07

**Authors:** Mohammad Rocky Khan Chowdhury, Md. Shafiur Rahman, Md. Mobarak Hossain Khan

**Affiliations:** 1 Department of Public Health, Faculty of Health Science, First Capital University of Bangladesh, Chuadanga, Bangladesh; 2 Department of Global Health Policy, School of International Health, University of Tokyo, Tokyo, Japan; 3 Department of Public Health Medicine, School of Public Health, Bielefeld University, Bielefeld, Germany; 4 Department of Public Health, College of Applied Medical Sciences, King Faisal University, Hofuf, Saudi Arabia

**Keywords:** Complementary feeding, Children, Dimension index, Determinants, Bangladesh

## Abstract

**Background:**

Information concerning complementary feeding (CF) practice during infancy and early childhood is still scarce in Bangladesh. Therefore, this study aimed to estimate the level of CF among children of 6–23 months and identify individual, household and community level determinants in Bangladesh.

**Methods:**

Secondary data from the Bangladesh Demographic Health Survey (BDHS) 2011 was used. A total of 2,373 children aged 6–23 months were selected. A simplified index called “dimension index” was used to estimate the level of CF. The score of this index was used either as continuous or categorical dependent variables. The highest score based on dimension index is associated to an adequate CF. Statistical analyses and tests were guided by types of variables. Finally, multivariable logistic regression (binary and multinomial) analyses were performed to identify the significant determinants of CF.

**Results:**

The overall level of CF among children of 6–23 months was low. More than 90 % of children experienced either no (2.9 %) or inadequate CF (92.7 %). According to bivariable analyses, mean levels of CF as well as percentages of no/inadequate CF were significantly lower among children of the youngest age group, uneducated parents, unemployed/laborer fathers, socio-economically poor families, food insecure families and rural areas. No weekly exposure to mass media (namely watching TV and reading newspapers/magazines) also revealed significant associations with CF. However, only few variables remained significant for adequate CF in the multivariable logistic regression analysis. For example, the likelihood of experiencing adequate CF was significantly lower among children of 6–11 months (OR: 0.22, 95 % CI: 0.10–0.47), children of illiterate fathers (OR: 0.32, 95 % CI: 0.11–0.95) and socio-economically middle-class families (OR: 0.28, 95 % CI: 0.09–0.86) as compared to their reference categories.

**Conclusion:**

A high level of inadequate CF leading to malnutrition may cause serious health problems among children of 6–23 months in Bangladesh. Vulnerable groups of children (e.g., the children aged 6 to 11 months and children of illiterate fathers), who received low levels of adequate CF, should be targeted by government and other stakeholders while developing strategies and interventions in order to improve overall situation of CF in Bangladesh.

## Background

Malnutrition refers to inadequate dietary intake, infectious disease, or a combination of both [1–3]. Worldwide about 2.3 million children’s deaths are attributed to malnutrition per year [4], which is nearly one-half of all child deaths [2]. The malnourished children are more likely to die from common childhood illnesses, such as, diarrhea, pneumonia, malaria, measles, and AIDS [5]. Malnutrition affects cognitive and educational development of millions of children, which cuts future earning by at least 20 % and reduces global economy substantially [4]. Some major causes of malnutrition include a lack of quality food, poor infant and child feeding and care practices, deficiencies of micronutrients such as vitamin A or zinc, and recurrent attack of infections, often intensified by intestinal parasites [6, 7]. Adequate complimentary feeding practice (CFP) can prevent up to 19 % of all childhood deaths in low-income countries [8]. Although Bangladesh has made magnificent progress in health and human development since its independence in 1971 [9, 10], this country still shows limited success in addressing the odds of child malnutrition. For instance, prevalence of under-five child malnutrition in Bangladesh is nearly 40 %, which causes nearly 60 % of under-five deaths there [11]. Inappropriate feeding practice could be considered as one of the profound causes of high under-five mortality in this country [12].

Proper feeding practices during infancy and early childhood are fundamental for normal growth, development, and survival of infants and children, particularly in developing countries [13–15]. South Asian countries including Bangladesh reveal the highest burden of childhood undernutrition due to unimproved feeding of children that causes faltered growth and development, and illnesses such as respiratory infections and diarrheal diseases [13, 16]. According to various studies, 6–23 months of age of a child is a “critical window” for transition of body and cognitive development [13, 17]. After 6 months of age, children need complementary food because breast milk or infant formula alone is no longer sufficient to maintain child’s growth [13, 17]. At this stage, children should be fed small quantities of nutritional solid and semisolid foods in addition to breast-feeding [17]. The World Health Organization (WHO) and the United Nations International Children’s Emergency Fund (UNICEF) have articulated a global strategy and formulated guidelines for complementary feeding (CF) of breastfed children [18]. Although appropriate CF among children aged 6–23 months brings numerous health benefits [13], inappropriate introduction of CF may increase risk of malnutrition among under-five children [19–22]. Levels of CF can be affected by numerous individual, household and community level factors [20–22]. Undernourished children are more likely to develop severe health hazards that impede body’s metabolism and retard utilization of immunity resulting from deficiencies in immune competence [23, 24].

Considering the limited number of studies in Bangladesh, this study first aimed to estimate levels of CF among children of 6 to 23 months using a composite dimension index and then to identify determinants of CF focusing on individual, household and community level factors. To our knowledge, no previous studies used a composite dimension index to measure the levels of CF in Bangladesh. Although dimension index is originally developed and used to calculate the Human Development Index (HDI) by the United Nations Development Programme (UNDP), it is also applied to address other public health issues [25, 26]. We used this index as an alternative of the available indices. As compared to this index (i.e., composite dimension index), other indices that quantified infant and young child feeding practice (IYCFP) in developing countries used limited number of variables [15, 27].

## Methods

### Ethical issue

#### Study design

Relevant data was extracted from the Bangladesh Demographic Health Survey (BDHS) 2011, which was a nationally representative cross-sectional survey. This survey was funded by the United States Agency for International Development (USAID), and conducted by the National Institute of Population Research and Training (NIPORT) under the Ministry of Health and Family Welfare, Bangladesh. All survey-related were implemented by a Bangladeshi research organization ‘Mitra and Associate’ with technical support from the ICF International of Calverton, Maryland, USA. According to their information, the 2011 BDHS was reviewed and approved by the ICF Macro Institutional Review Board (USA), which complies with all of the requirements of 45 CFR 46 “Protection of Human Subjects”. The 2011 Bangladesh DHS was also reviewed and approved by the National Research Ethics Committee of the Bangladesh Medical Research Council (Dhaka, Bangladesh). Informed consent was also obtained verbally from each participant (ever married women aged 15–49 years old) prior to subject enrollment. Since a significant part of the study sample was illiterate, verbal consent was the most suitable option to confirm participation. Since the BDHS included a sample of very young children (who were born either in 2009 or later) for data collection, mothers of this sample were also asked to provide verbal consent on behalf of their children. Each mother was asked to report about complementary foods that her child had consumed during the day or night preceding the interview. When mothers had more than one child in the study sample, they were asked to report about the youngest child living with them. Specifically, those mothers were asked to report about the number of solid or semi-solid complementary foods which were given to their children during the period.

### Setting

The BDHS 2011 sample was drawn from adults of selected households. The survey was undertaken in seven administrative regions (divisions): Southern region (Barisal), Southeastern region (Chittagong), Central region (Dhaka), Western region (Khulna), Midwestern region (Rajshahi), Northwestern region (Rangpur) and Eastern region (Sylhet) covering both rural and urban areas. In turn, each region (division) was divided into districts, and each district into upazilas (sub-districts). Rural areas in an upazila were divided into union parishads, and union parishads were further divided into mouzas. Urban areas in an upazila were divided into wards, and wards were subdivided into mahallas. Such classifications were taken into account to divide the country into rural and urban areas. Enumeration areas from the 2011 census were used as the Primary Sampling Units (PSUs) for survey. An enumeration area, which consists of about 100 households on an average, is equivalent to a mauza in rural areas and to a mohallah in urban areas. The survey is based on multistage stratified sampling techniques of households. At the first stage of sampling, 600 PSUs were selected (393 from rural area and 207 from urban areas). The resulting lists of households were used as sampling frames for the second stage of sampling. On an average, 120 households were selected from each PSU in the second stage, using an equal probability systematic sampling technique. Detailed description concerning sampling design and other related issues of the BDHS is available elsewhere [28].

### Selection of sample (inclusion and exclusion criteria)

**Fig. 1:**
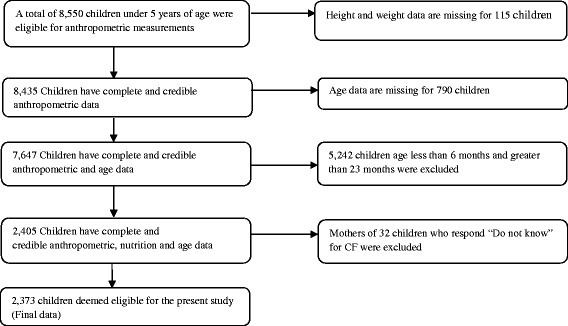
Schematic presentation of sample size selection

All children, born after January 2006 or later, were eligible for anthropometric (height and weight) measurements. All types of logistic supports were provided by the United Nations Children’s Emergency Fund (UNICEF). Several exclusion and inclusion criteria were used to get required sample. First, some criteria based on the World Health Organization (WHO) 2006 standards flag limits of z-scores were applied to measure implausible values of stunting, wasting and underweight. Generally a child is stunted, wasted, and underweight if the height-for-age z-scores (HAZ), weight-for-height z-scores (WHZ), and weight-for-age z-scores (WAZ) were less than 2 standard deviations (SDs) below the respective median of the WHO reference population, respectively [28]. For stunting, implausible values were defined as values of HAZ below −6 or above +6. Implausible values of wasting were defined as values of WHZ below −5 or above +5. Similarly, implausible values of underweight were the values of WAZ either below −6 or above +5. Second, from a total of 8,761 children below 5 years of age who were eligible for anthropometric measurements, completed data on height, weight and age were available only for 7,647 children. Third, all under-five children outside the range of 6–23 months were excluded from present analysis. Lastly, from a total of 2,405 children aged 6–23 months, 32 children were again excluded due to missing information (see Fig. 1). Therefore, our final sample for analysis was 2,373 children aged 6–23 months. Among them 37.9 % were stunted, 15.2 % were wasted and 31.7 % were underweight. The mean age, weight, height and hemoglobin level of the children were 14.19 (± 5.17) months, 8.30 (± 1.51) kg, 72.72 (± 6.51) cm, and 10.20 (± 1.24) g/dl, respectively.

### Covariates

Covariates were classified into three groups: individual-, household-, and community-level characteristics. Individual level characteristics were: age of child (6–11 months, 12–17 months, 18–23 months); sex of child (male, female); mother’s education (illiterate, literate); father’s education (illiterate, literate); father’s employment status (currently unemployed composed of unemployed, students etc.; labours composed of farmer, agricultural worker, fisherman and rickshaw etc.; service holders composed of doctor, lawyer, accountant, teacher etc.; businessman). Household socio-economic status based on wealth index (poor, middle, rich), mass media exposure through television, radio and newspaper/magazine (yes: defined as exposure to all medias at least once a week, no); food insecurity (yes, no) were considered as the household level characteristics. Community level characteristics were represented by the place of residence (urban, rural) and region of residence (Southern, Southeastern, Central region, Western region, Midwestern region, Northwestern and Eastern region).

### Household food security indicators

Five household food security indicators were selected using the Household Food Insecurity Access Scale. The technical working group of the BDHS 2011 systematically reviewed and modified the indicators to suit Bangladesh. The questions used were: (1) “In the past 12 months, did you have 3 square (‘full-stomach’) meals a day?”; (2) “In the past 12 months, did you have to skip entire meals because there was not enough food?”; (3) “In the past 12 months, did you have less food in a meal because there was not enough food?”; (4) “In the past 12 months, did you or any of your family members eat wheat or another grain in place of rice?”; and (5) “In the past 12 months, did you ask for food from relatives or neighbors to make a meal?” Each indicator had four options to select the best answer: never, rarely (1–6 times in the past 12 months), sometimes (7–12 times in the past 12 months), and often (few times each month). First, answers of these questions were numerically coded and then simply added to compute a total score for each ever-married women. For the first question, coded values were 3 = never, 2 = rarely, 1 = sometimes and 0 = often. Opposite coding were used for remaining questions with never = 0, rarely = 1, sometimes = 2 and Often = 3. A household was classified as food insecure when a family reported any of three options within the recall period of past month: never = 3, rarely = 2 and sometimes =1 for the first question and rarely = 1, sometimes =2 and often =3 for remaining four questions. According to our analysis, the total score ranged from 0 to 15. Any household with a total of score of 0 was classified as food secure family. In contrast, any household with a total scores between 1 and 15 was considered as food insecure family. To facilitate analysis, a composite score ranging from a minimum of “0” to a maximum of “15,” which was later classified as 2 categories, food secure (0), food insecure (>0) [28]. Wealth index was used to measure household socioeconomic status. This index was constructed based on household assets, including ownership of durable goods (such as televisions and bicycles) and dwelling characteristics (such as source of drinking water, sanitation facilities, and construction materials). The techniques of principal components analyses were used to assign individual household wealth scores. These weighted values were then summed and rescaled to range from 0–1 and later classified as poor, middle and rich [29].

### Levels of CF and outcomes

**Fig. 2:**
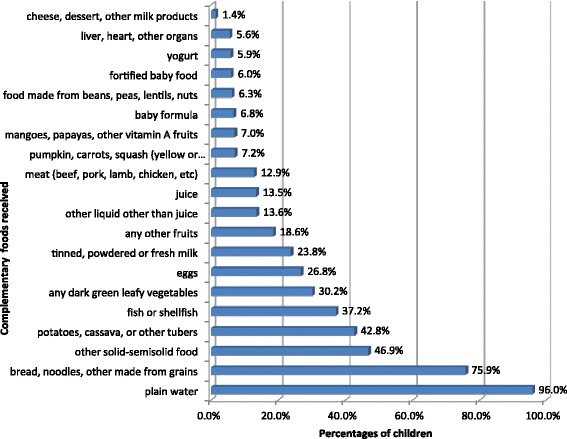
Percentage of complementary foods received by 6–23 months children

A child needs a variety of foods that provide adequate energy, protein, and micronutrients to meet nutritional needs [29]. To assess levels of CF among children, the BDHS 2011 survey included 20 questions for mothers. These were as follows: Did you give your child plain water? Did you give your child juice? Did you give your child tinned, powdered or fresh milk? Did you give your child baby formula? Did you give your child fortified baby food? Did you give your child other liquid? Did you give your child bread, noodles, other made from grains? Did you give your child potatoes, cassava, or other tubers? Did you give your child eggs? Did you give your child meat (beef, pork, lamb, chicken, etc.)? Did you give your child pumpkin, carrots, squash (yellow or orange inside)? Did you give your child any dark green leafy vegetables? Did you give your child mangoes, papayas, other vitamin A fruits? Did you give your child any other fruits? Did you give your child liver, heart, other organs? Did you give your child fish or shellfish? Did you give your child food made from beans, peas, lentils, nuts? Did you give your child cheese, yogurt and other milk products? Did you give your child other solid-semisolid food? Did you give your child yogurt? For each question, binary responses were made as 1 = yes (practice) and 0 = no (not practice). The percentages of children received these food items were presented in Fig. 2 for descriptive purpose. Thereafter, the formula of “dimension index” (given below) was used in accordance with the construction method of the Human Development Index (HDI) to estimate the level of CF [30]. Firstly, the Cronbach’s alpha coefficient was used to evaluate internal reliability of 20 indicators. According to our analysis, the Cronbach’s alpha coefficient was 0.701, suggesting a high internal consistency. The index was then constructed using the sums of weighted binary input variables where maximum and minimum values were also chosen for underlying dimension. The index is defined as:

$$ \mathrm{Dimension}\ \mathrm{Index}=\frac{\mathrm{Actual}\ \mathrm{value}-\mathrm{Minimum}\ \mathrm{value}}{\mathrm{Maximum}\ \mathrm{value}-\mathrm{Minimum}\ \mathrm{value}} $$

Where, actual value indicated a sum score of 20 binary indicators for each respondent, maximum value was 20 and minimum value was 0. This index was expressed into a unit-free index between 0 and 1 [30]. The individual score of CF (based on dimension index) was converted into percentage by multiplying 100. These scores were then divided into two (binary) groups, where individuals with dimension score of exactly 0 % belonged to “no CF” group and individuals with scores from 1 to 100 % belonged to “CF” group. The CF scores were again divided into three (multiple) categories: no CF (0 %), inadequate CF (1-49 %) and adequate CF (≥50 %). Briefly, both binary (no CF, CF) and multiple (no CF, inadequate CF, adequate CF) variables were used as dependent variables in multivariable logistic regression analyses.

### Statistical analysis

The continuous scores of CF based on the dimension index were tested using ANOVA-test and *t*-test. These tests were used to check whether differences of CF among various categories of each independent variable were significant. Contingency analysis was used to test bivariate associations between multiple dependent variable and selected factors by applying the Chi-square (χ^2^) test. In order to examine associations of selected factors with CF, first multivariable binary logistic regression was used for binary dependent variable and then multivariable multinomial logistic regression was applied for multiple dependent variables with three categories. Statistical significance was accepted at *P* < 0.05. All statistical analyses were carried out using SPSS software (version 20.0).

## Results

The detailed background characteristics of the respondents are given in Table 1. About half of the children were male and seven out of 10 children were from rural areas. Around 36 % children belonged to the age group of 6–11 months. Similarly, around 60 % children were from rich families. Illiteracy rate was 16.0 % among mothers and 25.5 % among fathers. The major category of father’s employment was labor (70.2 %). About two-fifth (39.2 %) of mothers did not watch TV weekly and two-third (65.3 %) children belonged to food insecure families.

**Table 1:** Mean level of complementary feeding

Variables	Numbers (%)	Mean (± SD)	Standard Error (SE)	*P* values
Children age (months)				
6–11	853 (35.9)	19.1 (± 11.7)	0.40	<0.001^a^
12–17	800 (33.7)	26.2 (± 11.8)	0.42	
18–23	720 (30.3)	28.0 (± 12.2)	0.45	
Sex of child				
Male	1199 (50.5)	23.9 (± 12.4)	0.36	0.332^b^
Female	1174 (49.5)	24.5 (± 12.5)	0.36	
Mother’s education				
Illiterate	380 (16.0)	18.8 (± 9.97)	0.51	<0.001^b^
Literate	1993 (84.0)	25.2 (± 12.7)	0.28	
Father’s education				
Illiterate	605 (25.5)	19.8 (± 10.7)	0.43	<0.001^b^
Literate	1768 (74.5)	25.7 (± 12.7)	0.30	
Father’s employment status				
Currently unemployed	53 (2.2)	22.6 (± 13.9)	1.91	<0.001^a^
Labours	1665 (70.2)	23.3 (± 11.8)	0.29	
Service holders	509 (21.4)	26.1 (± 13.2)	0.59	
Businessmen	146 (6.2)	28.8 (± 14.5)	1.20	
Socioeconomic status				
Poor	497 (20.9)	19.6 (± 10.6)	0.47	<0.001^a^
Middle	456 (19.2)	21.4 (± 11.1)	0.52	
Rich	1420 (59.8)	26.7 (± 12.8)	0.34	
Watch television				
No	930 (39.2)	20.7 (± 11.1)	0.36	<0.001^b^
Yes	1443 (60.8)	26.5 (± 12.8)	0.34	
Listen to radio				
No	2168 (91.4)	24.1 (± 12.5)	0.27	0.179
Yes	205 (8.6)	25.3 (± 12.8)	0.89	
Read newspapers/magazine				
No	1969 (83.0)	22.9 (± 11.8)	0.27	<0.001^b^
Yes	404 (17.0)	30.4 (± 13.7)	0.68	
Food insecurity				
No	1550 (65.3)	25.3 (± 12.9)	0.33	<0.001^b^
Yes	823 (34.7)	22.2 (± 11.3)	0.40	
Place of residence				
Urban	729 (30.7)	27.4 (± 13.4)	0.49	<0.001^b^
Rural	1644 (69.3)	22.8 (± 11.8)	0.29	
Region of residence				
Southern	266 (11.2)	23.7 (± 12.5)	0.76	<0.001^a^
Southeastern	499 (21.0)	22.3 (± 12.9)	0.58	
Central	378 (15.9)	23.6 (± 12.7)	0.66	
Western	269 (11.3)	28.8 (± 11.4)	0.69	
Mid-western	308 (13.0)	26.2 (± 11.8)	0.67	
Northwestern	299 (12.6)	25.9 (± 11.2)	0.65	
Eastern	354 (14.9)	21.1 (± 12.6)	0.67	
Total	2373 (100.0)	24.2 (± 12.5)	0.26	

*SD* Standard deviation, *SE* Standard error

^a^ANOVA test

^b^T-test

### Mean level of CF based on dimension index

The mean level of CF was 24.2 %, which was significantly lower among the children aged 6–11 months (19.1 %) (*p* < 0.001), illiterate mother (18.8 %) (*p* < 0.001), illiterate father (19.8 %) (*p* < 0.001) and children of currently unemployed fathers (22.6 %) (*p* < 0.001) (Table 1). Children belonging to the poorest socio-economic group (19.6 %) (*p* < 0.001), no exposure to television (20.7 %) (*p* < 0.001) and newspapers (22.9 %) (*p* < 0.001), food insecurity (22.2 %) (*p* < 0.001) and rural area (20.70 %) (*p* < 0.001) also revealed significantly lower CF scores. Moreover, the mean level of CF varied significantly among various geographical regions with the lowest level in southeastern region.

### Bivariate association of CF with multilevel independent variables

According to the categories of dimension index, more than 90 % children received inadequate complementary foods and almost 3.0 % children did not receive any complimentary foods. Children age, mother’s education, father’s education, father’s employment status, socio-economic status, mass media exposures (television and newspaper/magazine), food insecurity and place of residence were significantly associated with CF categories (Table 2). For instance, the percentage of adequate CF was significantly lower among younger group of children (2.5 %) (*p* < 0.001), among children of illiterate mother (1.8 %) (*p* = 0.028) and illiterate father (1.0 %) (*p* < 0.001). Similarly, the percentage of adequate CF was significantly lower among the children of those families, who belonged to the groups of socio-economically poor (1.6 %) (*p* < 0.001), no mass media exposure (*p* < 0.001), food insecurity (2.6 %) (*p* < 0.001) and rural area (3.0 %) (*p* < 0.001).

**Table 2:** Associations between selected factors and complementary feeding

Variables	Complementary feeding (CF)			*P* values
	No n (%)	Inadequate CF n (%)	Adequate CF n (%)	
Children age (months)				
6–11	41 (4.8 %)	791 (92.7 %)	21 (2.5 %)	<0.001
12–17	8 (1.0 %)	753 (94.1 %)	39 (4.9 %)	
18–23	19 (2.6 %)	656 (91.1 %)	45 (6.2 %)	
Sex of child				
Male	36 (3.0 %)	1109 (92.5 %)	54 (4.5 %)	0.903
Female	32 (2.7 %)	1091 (92.9 %)	51 (4.3 %)	
Mother’s education				
Illiterate	11 (2.9 %)	362 (95.3 %)	7 (1.8 %)	0.028
Literate	57 (2.9 %)	1838 (92.2 %)	98 (4.9 %)	
Father’s education				
Illiterate	19 (3.1 %)	580 (95.9 %)	6 (1.0 %)	<0.001
Literate	49 (2.8 %)	1620 (91.6 %)	99 (5.6 %)	
Father’s employment status				
Currently unemployed	3 (5.7 %)	48 (90.6 %)	2 (3.8 %)	<0.001
Labours	45 (2.7 %)	1568 (94.2 %)	52 (3.1 %)	
Service holders	17 (3.3 %)	457 (89.8 %)	35 (6.9 %)	
Businessmen	3 (2.1 %)	127 (87.0 %)	16 (11.0 %)	
Socioeconomic status				
Poor	15 (3.0 %)	474 (95.4 %)	8 (1.6 %)	<0.001
Middle	17 (3.7 %)	433 (95.0 %)	6 (1.3 %)	
Rich	36 (2.5 %)	1293 (91.1 %)	91 (6.4 %)	
Watch Television				
No	31 (3.3 %)	880 (94.6 %)	19 (2.0 %)	<0.001
Yes	37 (2.6 %)	1320 (91.5 %)	86 (6.0 %)	
Listen radio				
No	61 (2.8 %)	2013 (92.9 %)	94 (4.3 %)	0.691
Yes	7 (3.4 %)	187 (91.2 %)	11 (5.4 %)	
Read newspapers/magazine w				
No	59 (3.0 %)	1850 (94.0 %)	60 (3.0 %)	<0.001
Yes	9 (2.2 %)	350 (86.6 %)	45 (11.1 %)	
Food insecurity				
No	45 (2.9 %)	1421 (91.7 %)	84 (5.4 %)	0.005
Yes	23 (2.8 %)	779 (94.7 %)	21 (2.6 %)	
Place of residence				
Urban	14 (1.9 %)	660 (90.5 %)	55 (7.5 %)	<0.001
Rural	54 (3.3 %)	1540 (93.7 %)	50 (3.0 %)	
Region of residence				
Southern	4 (1.5 %)	252 (94.7 %)	10 (3.8 %)	0.398
Southeastern	22 (4.4 %)	451 (90.4 %)	26 (5.2 %)	
Central	7 (1.9 %)	354 (93.7 %)	17 (4.5 %)	
Western	6 (2.2 %)	248 (92.2 %)	15 (5.6 %)	
Mid-western	8 (2.6 %)	285 (92.5 %)	15 (4.9 %)	
Northwestern	9 (3.0 %)	278 (93.0 %)	12 (4.0 %)	
Eastern	12 (3.4 %)	332 (93.8 %)	10 (2.8 %)	
Total	68 (2.9 %)	2200 (92.7 %)	105 (4.4 %)	

### Multivariable association of CF with multilevel independent variables

The results of binary logistic regression were presented first under the dichotomous dependent variable “any CF”. According to these results (Table 3), children of 6–11 months of age were less likely to receive any CF (OR: 0.55, 95 % CI: 0.32–0.96) as compared to the group of 18–23 months. In contrast, children of 12–17 months of age were more likely to receive any CF (OR: 2.63, 95 % CI: 1.14–6.07). Other variables were not significantly associated with the dependent variable “any feeding”.

**Table 3:** Results of multivariable logistic regression analysis

Variables	Complementary feeding(CF)					
	Any CF (yes/no)		Inadequate CF		Adequate CF	
	Adjusted OR (95 % CI)	*P* values	Adjusted OR (95 % CI)	*P* values	Adjusted OR (95 % CI)	*P* values
Children age (months)						
6–11	0.55 (0.32–0.96)	<0.001	0.57 (0.33–1.00)	0.050	0.22 (0.10–0.47)	<0.001
12–17	2.63 (1.14–6.07)	0.037	2.68 (1.16–6.19)	0.021	1.84 (0.72–4.71)	0.206
18–23 (ref.)	1.00		1.00		1.00	
Sex of child						
Male	0.92 (0.56–1.49)	0.725	0.91 (0.56–1.50)	0.719	1.01 (0.54–1.89)	0.978
Female (ref.)	1.00		1.00		1.00	
Mother’s education						
Illiterate	1.11 (0.52–2.37)	0.779	1.11 (0.52–2.35)	0.792	1.48 (0.46–4.69)	0.509
Literate (ref.)	1.00		1.00		1.00	
Father’s education						
Illiterate	1.01 (0.54–1.89)	0.981	1.03 (0.55–1.92)	0.934	0.32 (0.11–0.95)	0.040
Literate (ref.)	1.00		1.00		1.00	
Father’s employment status						
Currently unemployed	0.42 (0.08–2.25)	0.313	0.44 (0.08–2.36)	0.341	0.23 (0.02–2.12)	0.193
Labours	0.94 (0.28–3.20)	0.918	0.98 (0.29–3.35)	0.976	0.49 (0.13–1.89)	0.297
Service holders	0.68 (0.19–2.40)	0.551	0.70 (0.20–2.48)	0.584	0.50 (0.13–2.01)	0.330
Businessman (ref.)	1.00		1.00		1.00	
Socioeconomic status						
Poor	0.85 (0.39–1.84)	0.539	0.86 (0.40–1.86)	0.704	0.55 (0.18–1.70)	0.299
Middle	0.69 (0.35–1.34)	0.687	0.70 (0.36–1.37)	0.297	0.28 (0.09–0.86)	0.025
Rich (ref.)	1.00		1.00		1.00	
Watch Television						
No	0.89 (0.51–1.57)	0.695	0.90 (0.51–1.58)	0.712	0.68 (0.31–1.49)	0.336
Yes (ref.)	1.00		1.00	.	1.00	
Listen radio						
No	1.24 (0.55–2.81)	0.607	1.24 (0.55–2.81)	0.608	1.21 (0.42–3.44)	0.722
Yes (ref.)	1.00		1.00		1.00	.
Read newspapers/magazine						
No	0.82 (0.38–1.77)	0.602	0.86 (0.40–1.85)	0.702	0.38 (0.16–0.92)	0.032
Yes (ref.)	1.00		1.00		1.00	
Food insecurity						
No	0.83 (0.47–1.46)	0.526	0.83 (0.47–1.46)	0.518	0.90 (0.42–1.95)	0.796
Yes (ref.)	1.00		1.00		1.00	
Place of residence						
Urban	1.43 (0.75–2.70)	0.275	1.40 (0.74–2.65)	0.300	1.93 (0.90–4.13)	0.090
Rural (ref.)	1.00		1.00		1.00	
Region of residence						
Southern	2.40 (0.75–7.70)	0.276	2.40 (0.75–7.68)	0.141	2.84 (0.65–12.38)	0.164
Southeastern	0.72 (0.34–1.49)	0.140	0.71 (0.34–1.47)	0.357	1.26 (0.44–3.58)	0.663
Central	1.67 (0.64–4.34)	0.380	1.65 (0.65–4.30)	0.304	2.42 (0.69–8.45)	0.165
Western	1.34 (0.49–3.73)	0.294	1.32 (0.48–3.66)	0.592	2.44 (0.66–9.01)	0.182
Mid–western	1.19 (0.47–3.01)	0.570	1.17 (0.47–2.97)	0.735	2.03 (0.59–6.99)	0.263
Northwestern	1.19 (0.48–2.92)	0.711	1.17 (0.48–2.88)	0.733	2.04 (0.58–7.13)	0.263
Eastern (ref.)	1.00		1.00		1.00	

*CI* 95 % confidence interval

According to the results of multinomial logistic regression analysis (presented under the categories of inadequate and adequate take ‘no CF’ as a reference in Table 3), children of illiterate fathers had significantly less likelihood of having adequate CF (OR: 0.32, 95 % CI: 0.11–0.95). Children from the socioeconomically middle class families were less likely to receive adequate CF (OR: 0.28, 95 % CI: 0.09–0.86) as compared to rich children. Children of families with no exposure to newspaper/magazine also revealed significantly less likelihood of receiving adequate CF (OR: 0.38, 95 % CI: 0.16–0.92) than reference category.

## Discussion

The study reveals a low level of CF (composed of no CF or inadequate CF) among children aged 6–23 months in Bangladesh. Several selected factors, such as, children’s age, mother’s education, father’s education, father’s employment status, socio-economic status, mass media exposure, food insecurity, place of residence and region of residence were significantly associated with CF. Our findings are consistent with the findings of a study in Bangladesh [31] and other South Asian countries such as India, Pakistan, Sri Lanka and Nepal [32–35]. The higher prevalence of undernutrion among under-five children in Bangladesh can be attributed to the poorly received complementary foods during 6–23 months of age period of children. Financial constraints, lack of knowledge and awareness regarding appropriate CF may influence the poor nutritional supplement among children [8, 36].

There are a number of studies that already assessed the child feeding practice. However, the number of variables to measure IYCFP (Infant and Young Child Feeding Practice) varied from study to study. For example, Srivastava (2007) constructed age specific IYCFP using five measurements, such as, continued breastfeeding, 24-h dietary diversity, frequency of feeding solids/semi-solid foods, psychosocial care during feeding and hygiene during preparation and feeding in India [15]. However, they found the significant interclass variations of level of CF based on some socio-economic variables, such as, sex of child, age of child, birth order, socio-economic status and mothers BMI [15]. They also found the association between nutritional status and IYCFP [15]. The findings were partially consistent with our study. A similar structure of indexing was also used to assess the child feeding practice in rural India and urban Madagascar [15, 27, 37]. According to our knowledge, no previous studies have addressed the level of CF using dimension index based on 20 complementary food items.

We found significant interclass variations of CF for different individual, household and community level variables. For instance, receiving complementary foods were significantly lower among children of younger age group (6–11 months) than older age group (18–23 months). Several studies reported that percentages of infant receiving complementary foods increase with age but did not show any significant variations [38–40]. The CF was also found significantly lower among children of illiterate parents, children of father professionally labour, socioeconomically poor families, children of families never exposed to mass media, food insecure families, and rural settlement. These findings were consistent with another study conducted in Nepal [40].

Proper and appropriate CF among children can be hampered due to various reasons such as poverty, urban-rural differential, gender discrimination, lack of parental education and health literacy, lack of women participation in household decision making processes, high vulnerability due to environmental condition and scarce information in academic syllabus [26, 41–48]. Although Bangladesh made remarkable progress in various sectors, still this country experiences substantial poverty, inequality and deprivation. More than 30 % of Bangladeshi population lives below the poverty line and a significant proportion of them live in extreme poverty, especially in the countryside [41, 42]. The UNICEF reported that in Bangladesh over 33 million children under 18 years of old, which account for around 56 % of the child population, are currently living below the international poverty line and around 57 % are deprived of adequate nutrition [43]. One of the important questions to mention here is that why did children from rich families get no CF or inadequate CF? Although research-based explanations are unknown to the authors, it may be related to the lack of knowledge of mothers or other caregivers of children. It may also be related to the customary practice of the societies. Bangladesh is one of the South Asian countries where female children experience higher mortality [46]. In this country, more attention is paid to male children in intra-family food distribution and healthcare. Such kinds of discrimination against female children can aggravate the situation of undernutrition and other health hazards among female children compare to male [45]. Regional variation also has been observed in terms of CF. Variations in CF may conceal important intra-regional differences due to diverse cultural norms and needs more investigation.

The multivariable logistic regression analyses identified several determinants such as children’s age, father’s educational status, socio-economic status and mass media exposure for CF. These factors were also identified as significant predictors by other studies, particularly for the initiation of CF [49–52] and nutritional disorder of children [23, 53, 54].

Although numerous policies and strategies have been issued in Bangladesh to improve the IYCFP, some challenges such as insufficient resources and lack of coordination among stakeholders are impeding their implementation and enforcement. Therefore, strengthening the existing strategies such as eradication of poverty through marginalized and vulnerable group development, empowering women to practice decision making autonomy and minimizing rural urban differential through planning and providing modern facilities are urgently necessary to improve the situation of CF in Bangladesh. Proper CF can also be ensured by undertaking social safety net programmes and community-based nutritional interventions, for example, food for education, food for work for slum dwellers, etc. [30]. The feeding practice of home-based foods with various textures should be encouraged [52]. Health promotion programmes through cross-collaboration among various organizations and behavioral change communication through nutritional education, particularly in slum and rural areas, which support and educate mothers, need to be developed for improving appropriate feeding practice to children [8, 55, 56]. An integrated nutrition-sensitive social protection system is to be recommended under the Bangladesh’s national protection structure “National Social Security Strategy” to support the most vulnerable citizens in order to improve the nutritional status in Bangladesh [45]. In addition, more efforts should be given to review existing nutritional interventions that target the IYCFP in Bangladesh.

This study has several strengths. The main strength of this study is the utilization of a large nationally representative recent data that covers both urban and rural areas. Other strengths may include the use of dimension index to interpret CF, which is very simple and informative. Since studies based on this index are still scarce, further reports to provide universally accepted cut-off points to define different groups of CF would be immensely useful for the purposes of comparison, monitoring, evaluation and advocacy. This study is not free from limitations. For instance, due to unavailability of recent data, BDHS 2011 data was used in this study that does not present the current nutritional status. The cross-sectional nature of the study limits us to assess the cause and effect relationships between selected factors and CF. Most of the food items were given to the children in last 24-h preceding the survey time, however, the children could occasionally receive some other foods but simply did not the previous day. The dietary data on children are subject to recall errors on the mother’s part. In addition, a mother may not be able to report fully on a child’s intake of food and liquids if the child was fed by other individuals during the period. All food items were treated equally may limit our study. Another limitation could be information bias, which may result from collecting information of self-reporting age, education, occupation, household assets as well as nutritional indicators.

### Conclusion

This study revealed a high percentage of inadequate CF among children that could be the cause of malnutrition. Although several individual, household and community factors, such as, children age, mother’s education, father’s education, father’s employment status, socio-economic status, mass media exposures, food insecurity and place of residence indicated significant association with the levels of CF, only few of them remained significant in multivariable analysis. To improve the overall condition of CF, some strategies could be developed based on significant factors. Particularly the vulnerable group of children (e.g., street children, children from slums etc.) who received a significantly lower level of adequate CF should be targeted by the government and other stakeholders while developing strategies and interventions to address the issue of child CF in Bangladesh. Moreover, longitudinal studies are recommended to assess the cause-effect relationships between plausible factors and CF in Bangladesh.

## Abbreviations

BDHS: Bangladesh demographic health survey
CF: Complementary feeding
IYCFP: Infant and young child feeding practice
NIPORT: National institute of population research and training
UNDP: United Nations Development Programme
UNICEF: United Nations International Children’s Emergency Fund
USAID: United States agency for international development
WHO: World Health Organization
